# Supplementary figures and images for: Whole genome screen reveals a novel relationship between Wolbachia levels and Drosophila host translation
Source: PLoS Pathog. 2018 Nov 13;14(11):e1007445. doi: 10.1371/journal.ppat.1007445 (PMC6258568; doi:10.1371/journal.ppat.1007445)

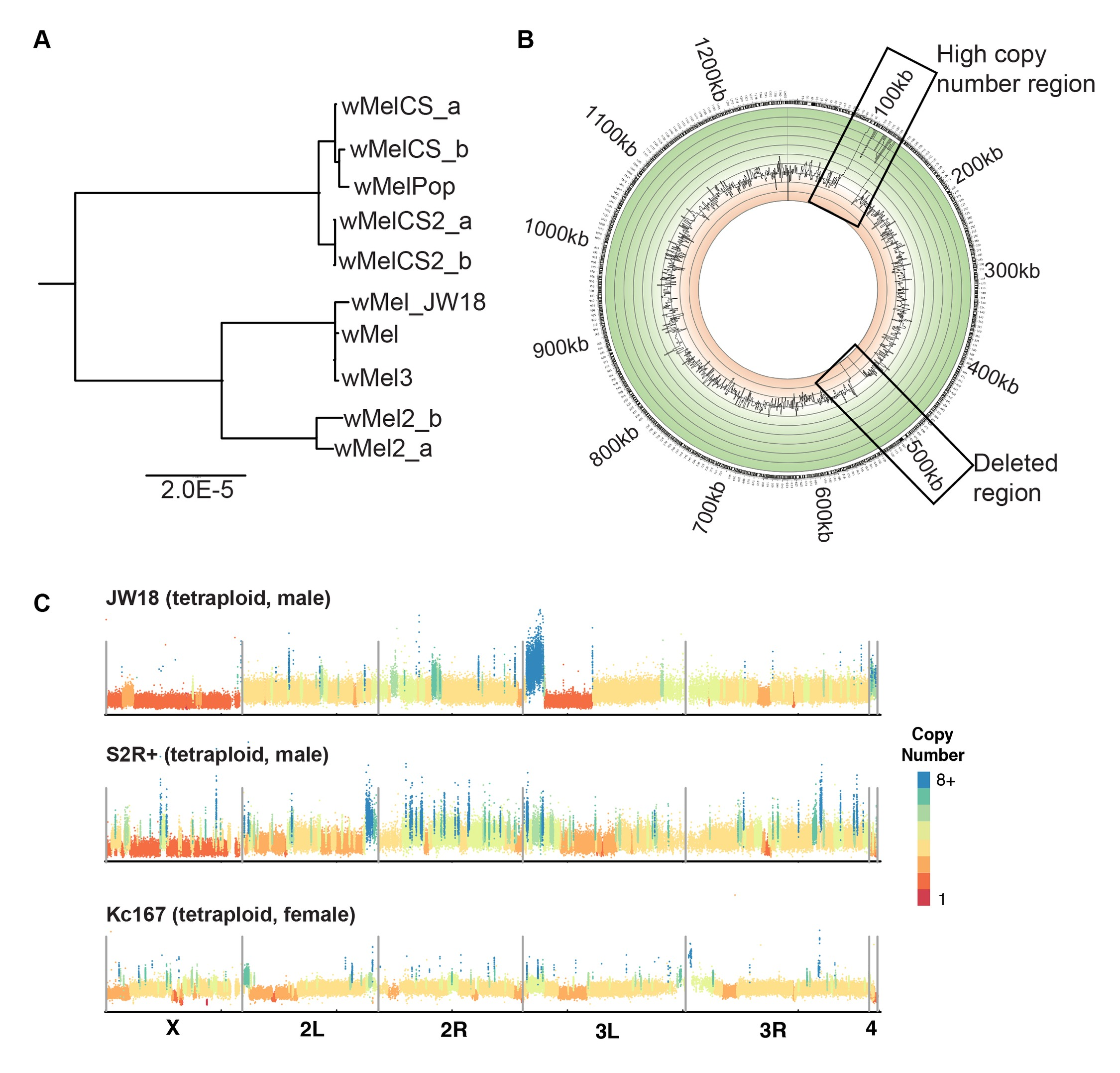

Supplement: S1 Fig — (A) Phylogenetic analysis of the Wolbachia strain in JW18 cells compared to previously sequenced strains [27]. (B) Genome-wide copy number analysis of Wolbachia strain in JW18 cells. (C) Comparison of genome-wide copy number variation of Wolbachia-infected JW18 Drosophila cell line with Wolbachia-free S2R+ and Kc167 Drosophila cell lines. Plots of mapped DNA read density along the genome. Deduced copy number is indicated by color (see key). Genome-wide copy number analysis is shown for three Drosophila cell lines: Wolbachia-infected JW18, S2R+, and Kc167. (TIF) [file ppat.1007445.s001.tif]

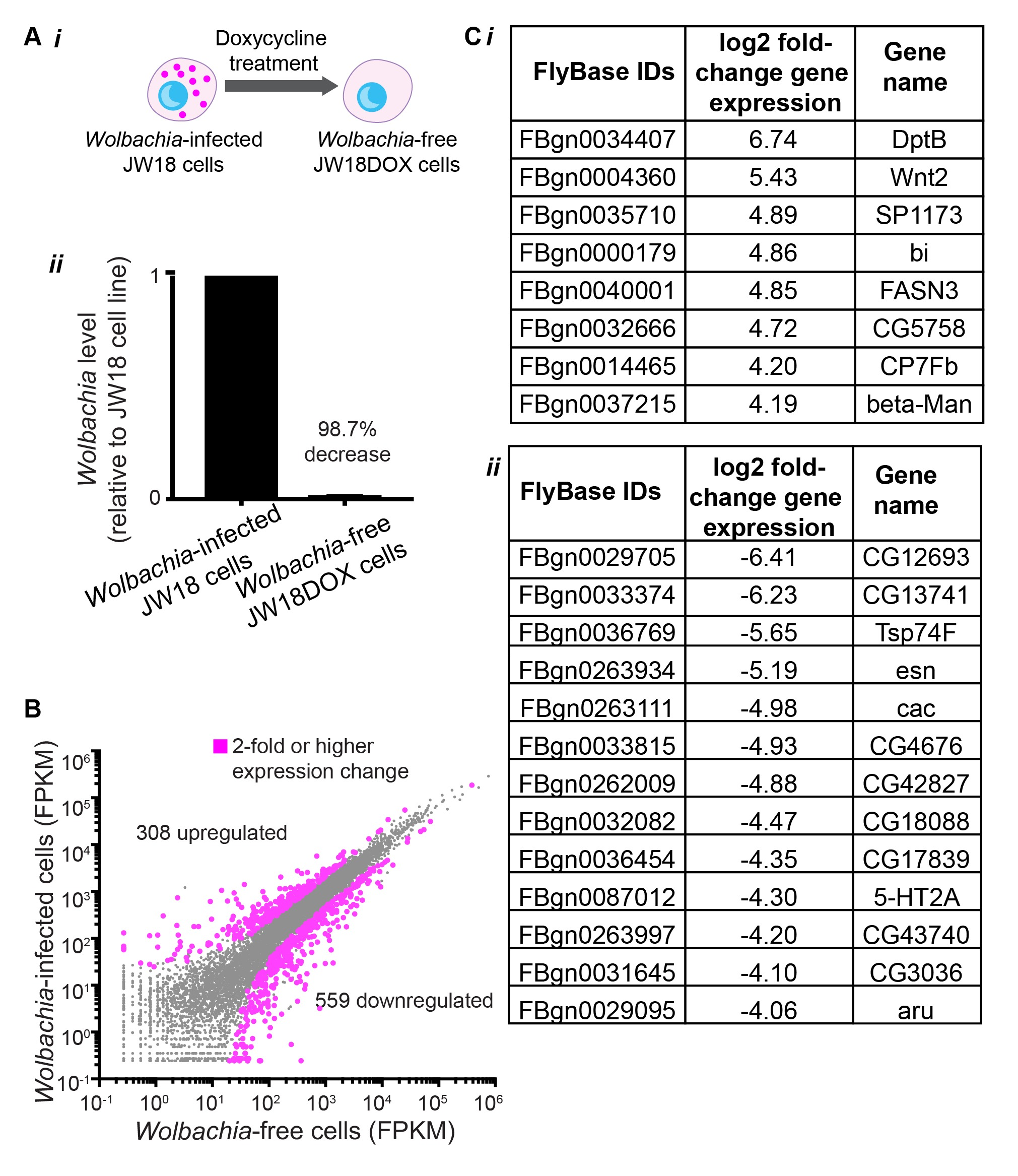

Supplement: S2 Fig — (A) (i) Wolbachia was removed from the JW18 cell line by treatment with doxycycline to generate a Wolbachia-free version of the cell line JW18DOX (conducted in W. Sullivan lab and obtained together with the infected cell lines at the beginning of our study). (A) (ii) Wolbachia infection in the JW18 cell line and the Wolbachia-free status of JW18DOX cell line confirmed by DNA qPCR assay (see methods). (B) Differential gene expression analysis from RNAseq data comparing changes in host gene expression in the presence (JW18) and absence (JW18DOX) of Wolbachia. (C) List of most highly upregulated (i) and most highly downregulated (ii) host genes in the presence of Wolbachia infection. (TIF) [file ppat.1007445.s002.tif]

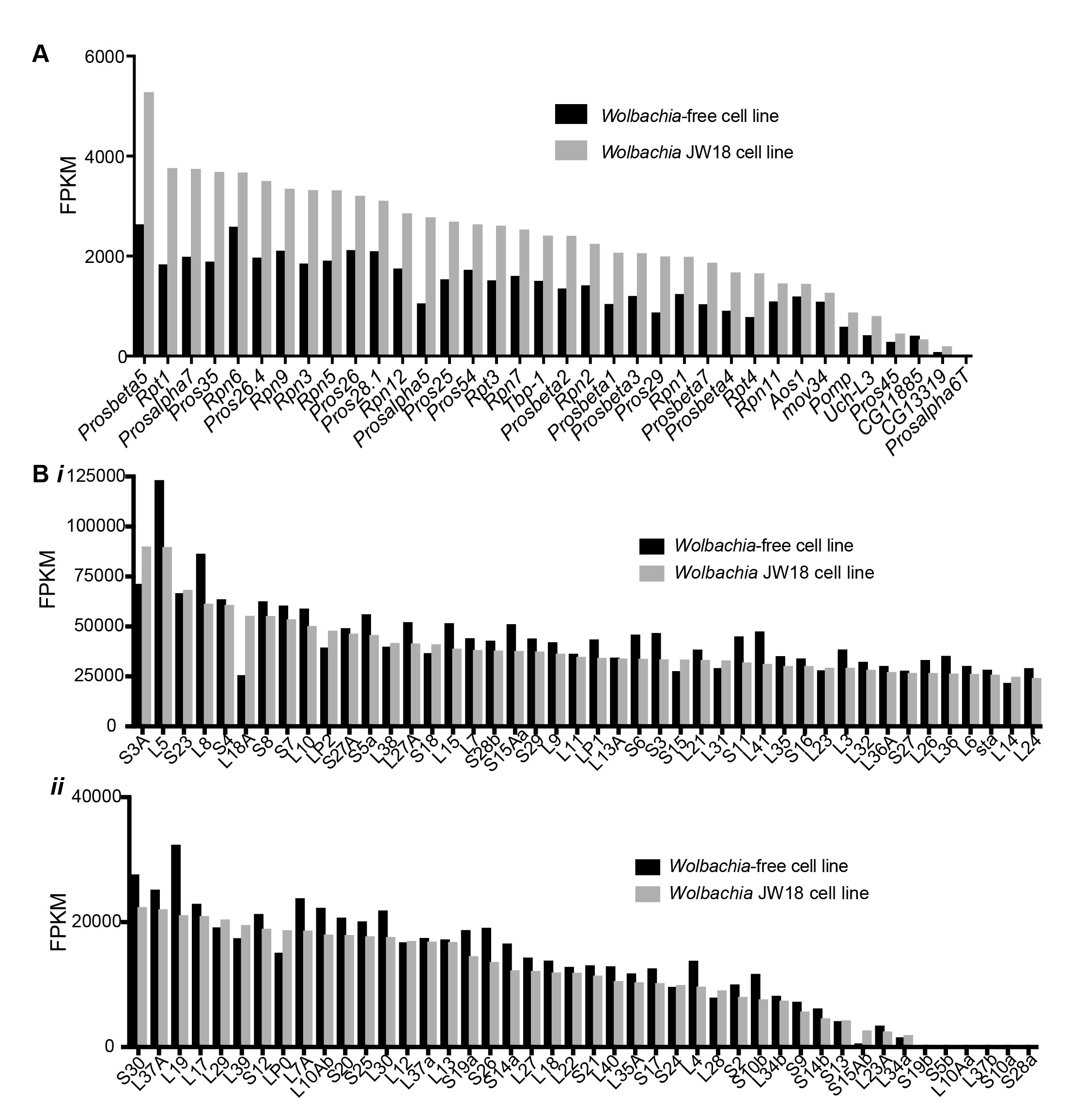

Supplement: S3 Fig — (A) The expression of genes encoding the host proteasome is upregulated in the presence of Wolbachia in the JW18 cell line. (B) The expression of host ribosome components is not different in the presence or absence of Wolbachia. (TIF) [file ppat.1007445.s003.tif]

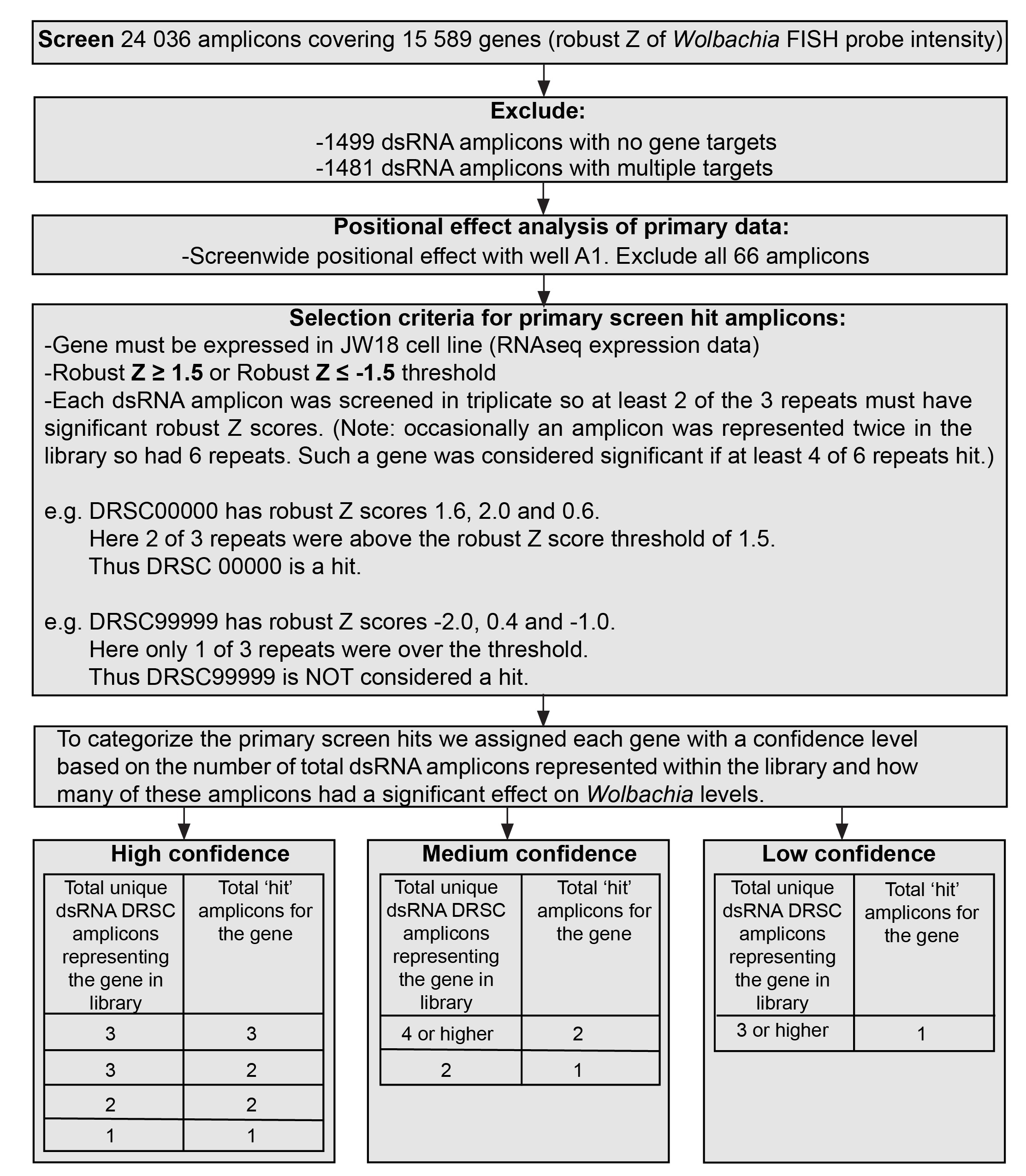

Supplement: S4 Fig — Quality control of primary data involved exclusion of outdated or non-specific dsRNA amplicons, positional effect analysis, and assessment of gene expression levels in JW18 cells. Thereafter, primary screen hits were selected based on threshold criteria and hits were categorized as low, medium, or high confidence. (TIF) [file ppat.1007445.s004.tif]

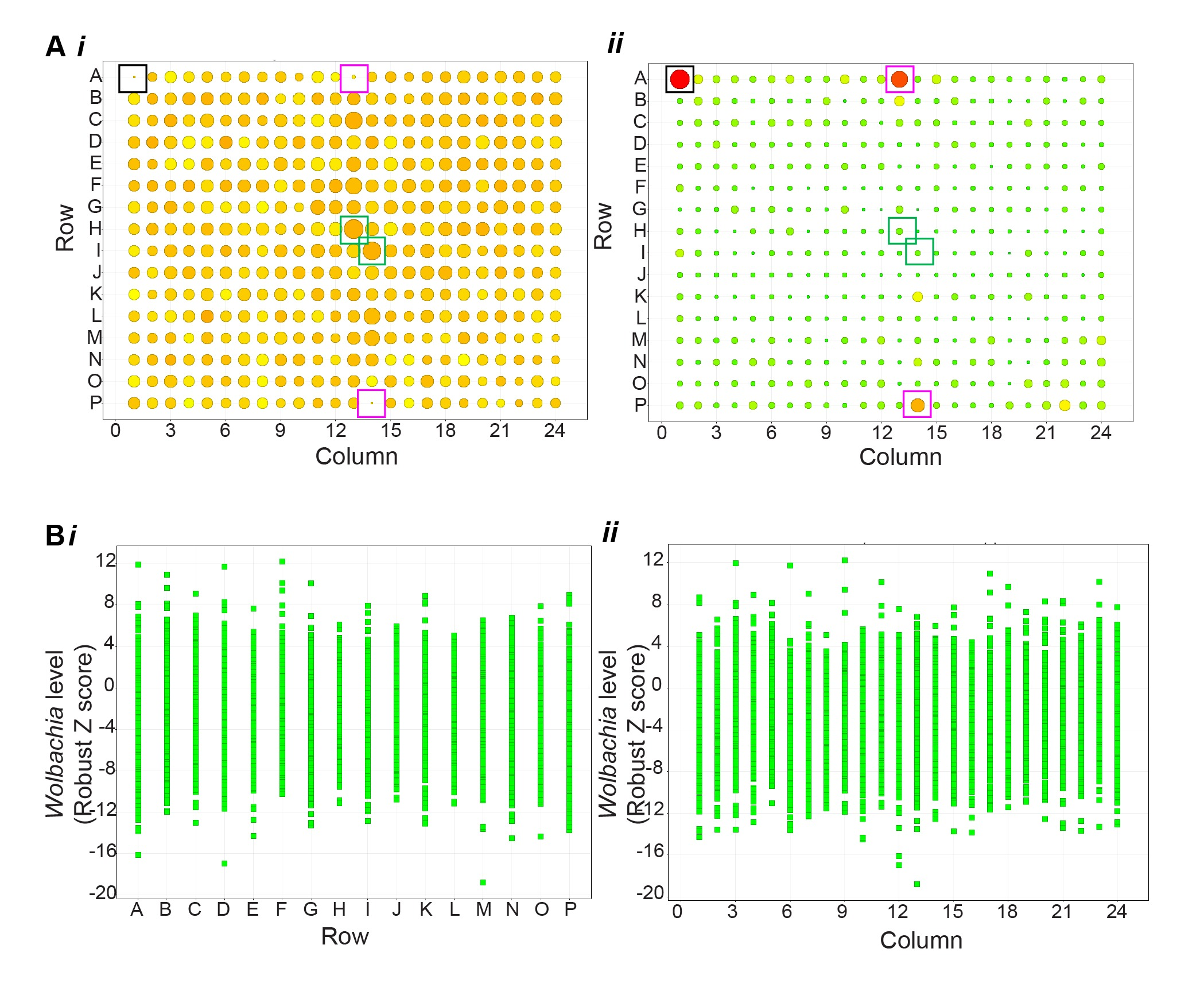

Supplement: S5 Fig — (A) Superimposed visual representations of the collective average robust Z scores (i) and standard deviations (ii) represented by dot sizes for each well position of all 198 384-well plates screened for the whole genome screen. RpL40 dsRNA control wells for increasing Wolbachia level are highlighted by the two green boxes. Doxycycline control wells for decreasing Wolbachia level are highlighted in two magenta boxes. Well A1 highlighted in the black box was excluded from further analysis because all 66 amplicons plated in well A1 across the screen had a very low robust Z score and the standard deviation was very high compared to all other well positions in the screen. (See S4 Table for list of amplicons seeded in well A1.) (B) Visual representation of Wolbachia levels in all wells grouped by row (i) and by column (ii). All visualization was done using Vortex software (Dotmatics, USA). (TIF) [file ppat.1007445.s005.tif]

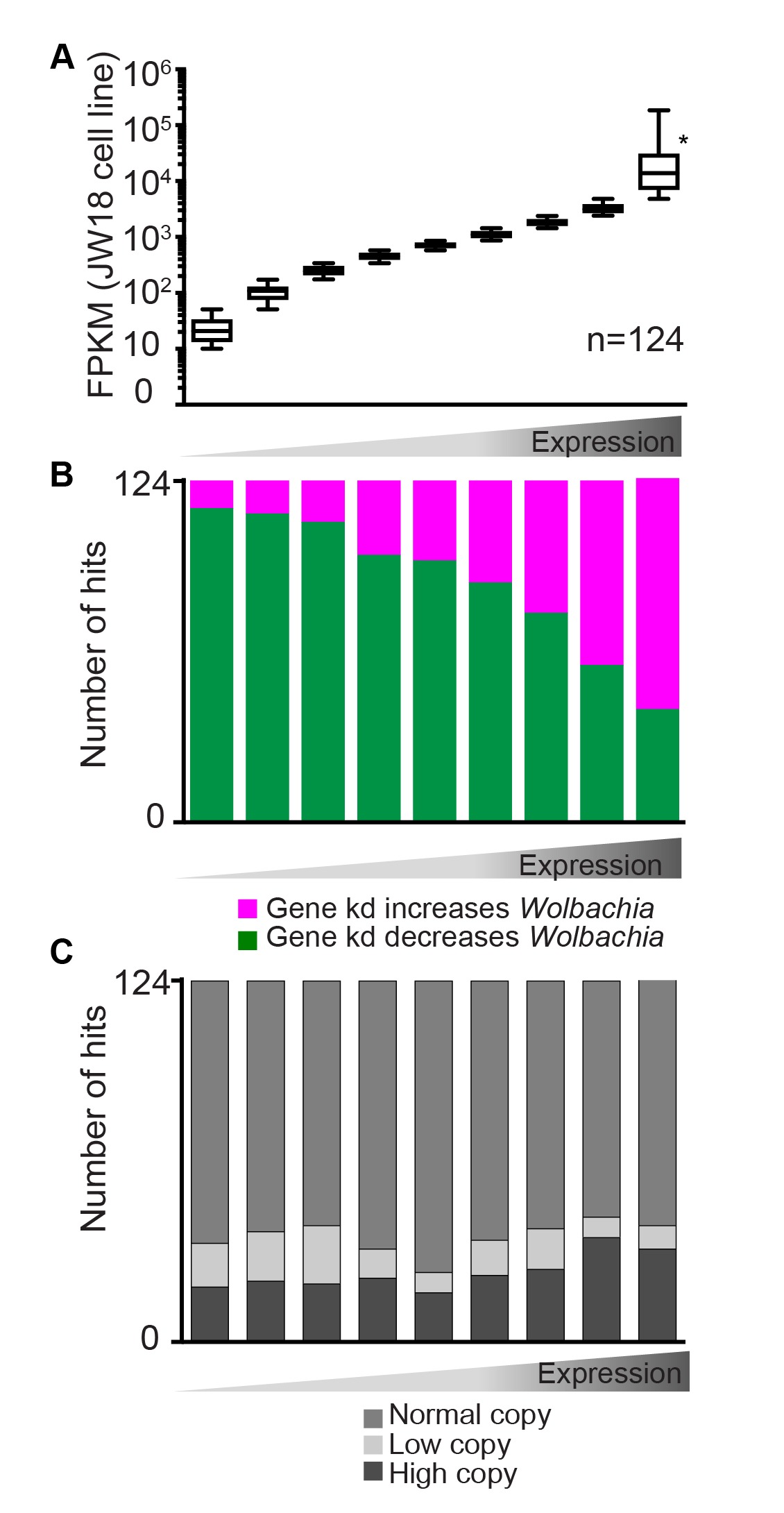

Supplement: S6 Fig — (A) 1117 primary hits binned into 9 bins of 124 genes each based on gene expression level from RNAseq data. * Note: Final bin only contains 123 genes. (B) Representation of the effect on Wolbachia level for primary hits within each bin (defined in A) including genes that increased (magenta) and decreased (magenta) Wolbachia upon RNAi knockdown. (C) Representation of gene DNA copy number variation of primary hits within the 9 bins (defined in A and B). (TIF) [file ppat.1007445.s006.tif]

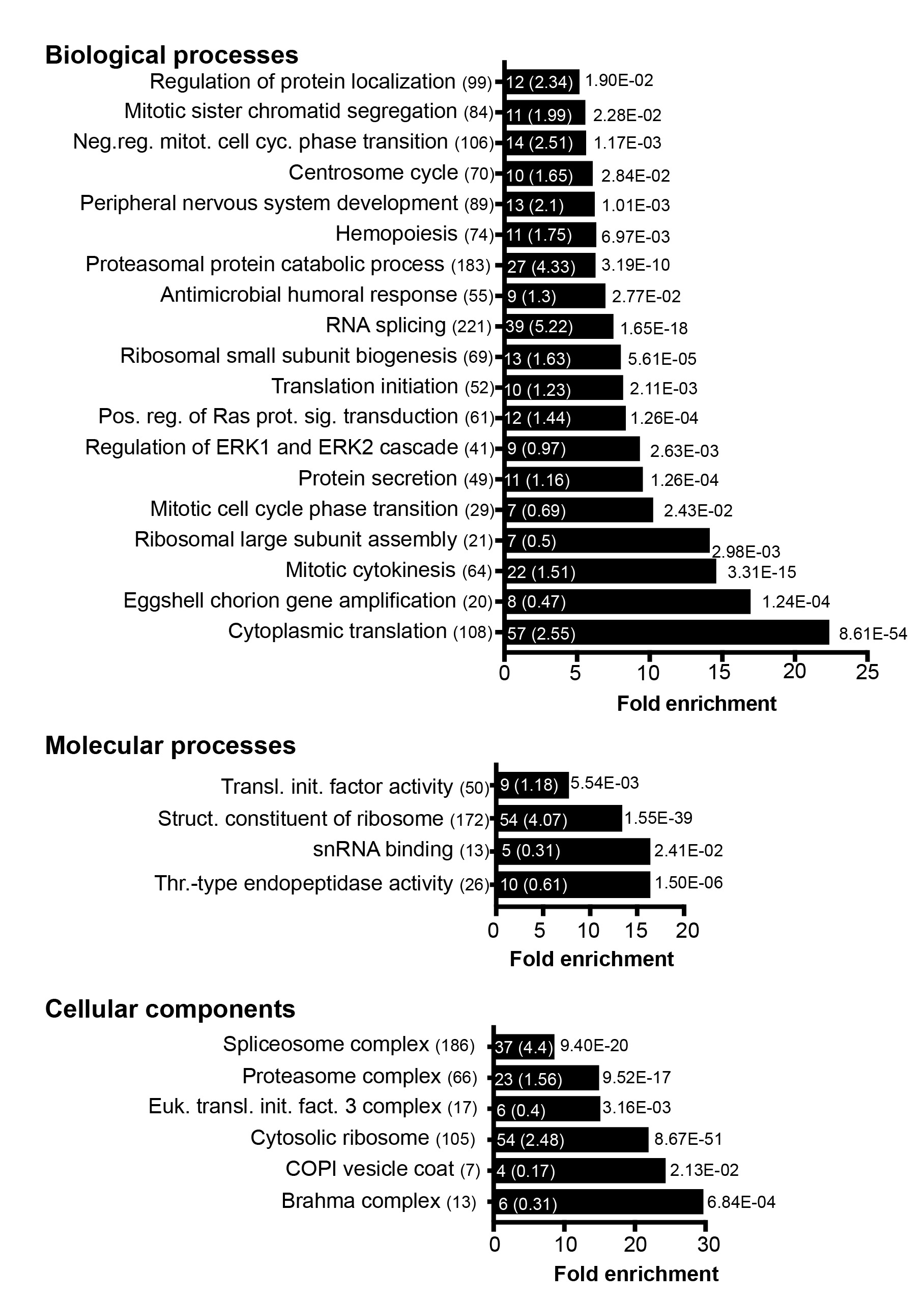

Supplement: S7 Fig — Primary screen hits that increased (329 genes) Wolbachia levels significantly upon RNAi knockdown were analyzed for gene ontology term enrichment in biological processes, molecular processes, and cellular components. Total genes for GO term in Drosophila melanogaster genome shown in brackets after term. Number of genes represented shown on the bar and the number of expected genes to hit by chance shown in brackets. p-values are represented after each bar. Note: No enrichment (enrichment score >5) of any terms for screen hits that decreased Wolbachia levels (788 genes) was found. Gene ontology analysis was performed using PANTHER Version 12.0 (release 2017-07-10). (TIF) [file ppat.1007445.s007.tif]

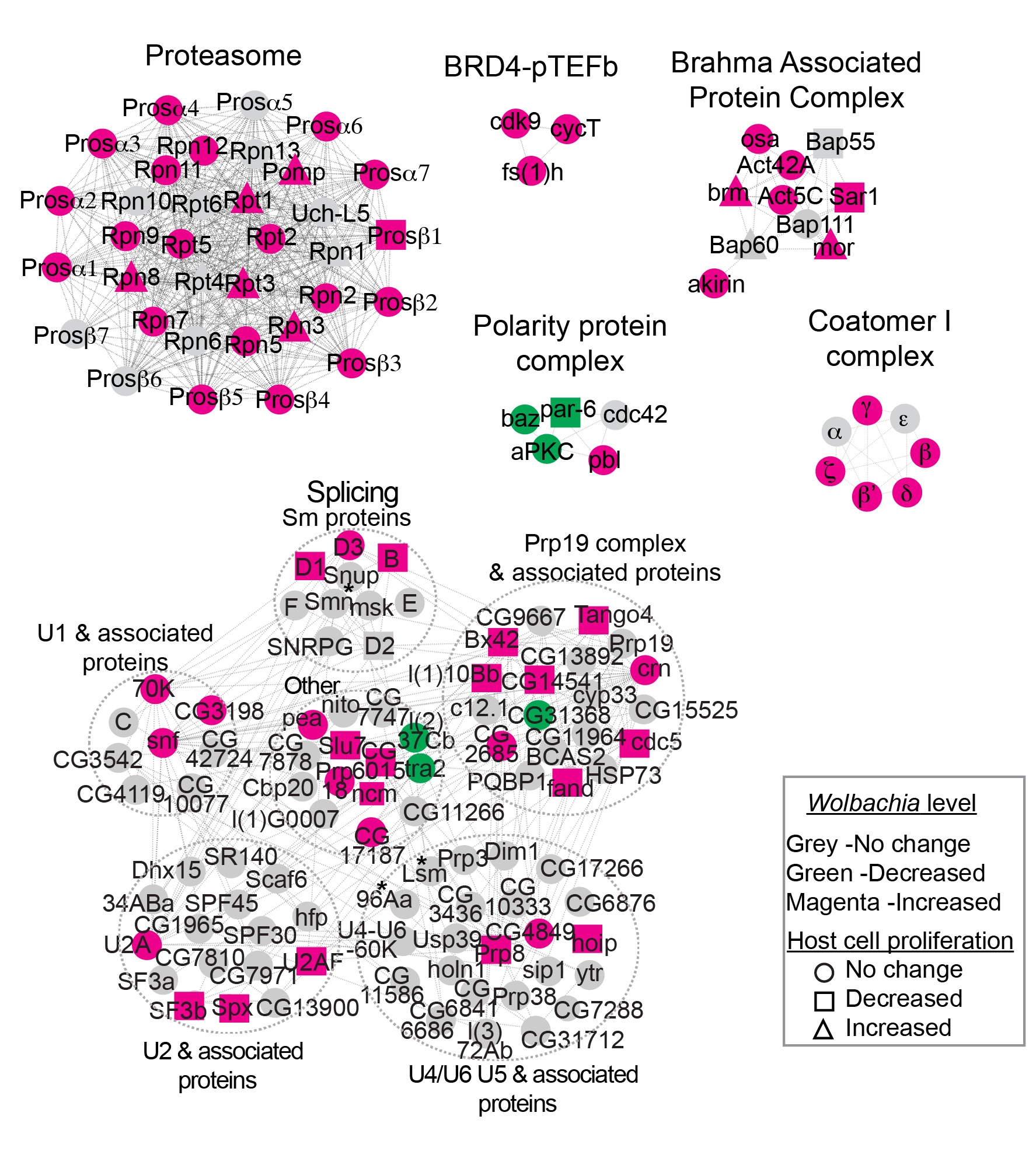

Supplement: S8 Fig — We identified the core ribosome (Fig 5), translation initiation complex (Fig 5), core proteasome, BRD4-pTEFb complex, Coatomer I complex, Brahma complex and components of the spliceosome as enriched for genes that increased Wolbachia levels in the primary screen. Three cell polarity proteins decreased Wolbachia levels in the primary screen. Changes in Wolbachia levels in the primary screen are indicated by color: increases (magenta), decreases (green), and no effect (grey). Changes in cell proliferation during the whole genome screen assay are indicated by icon shape: no change (circle), decrease (square), and increase (triangle). Note: These results represent the raw results from the screen prior to secondary validation. (TIF) [file ppat.1007445.s008.tif]

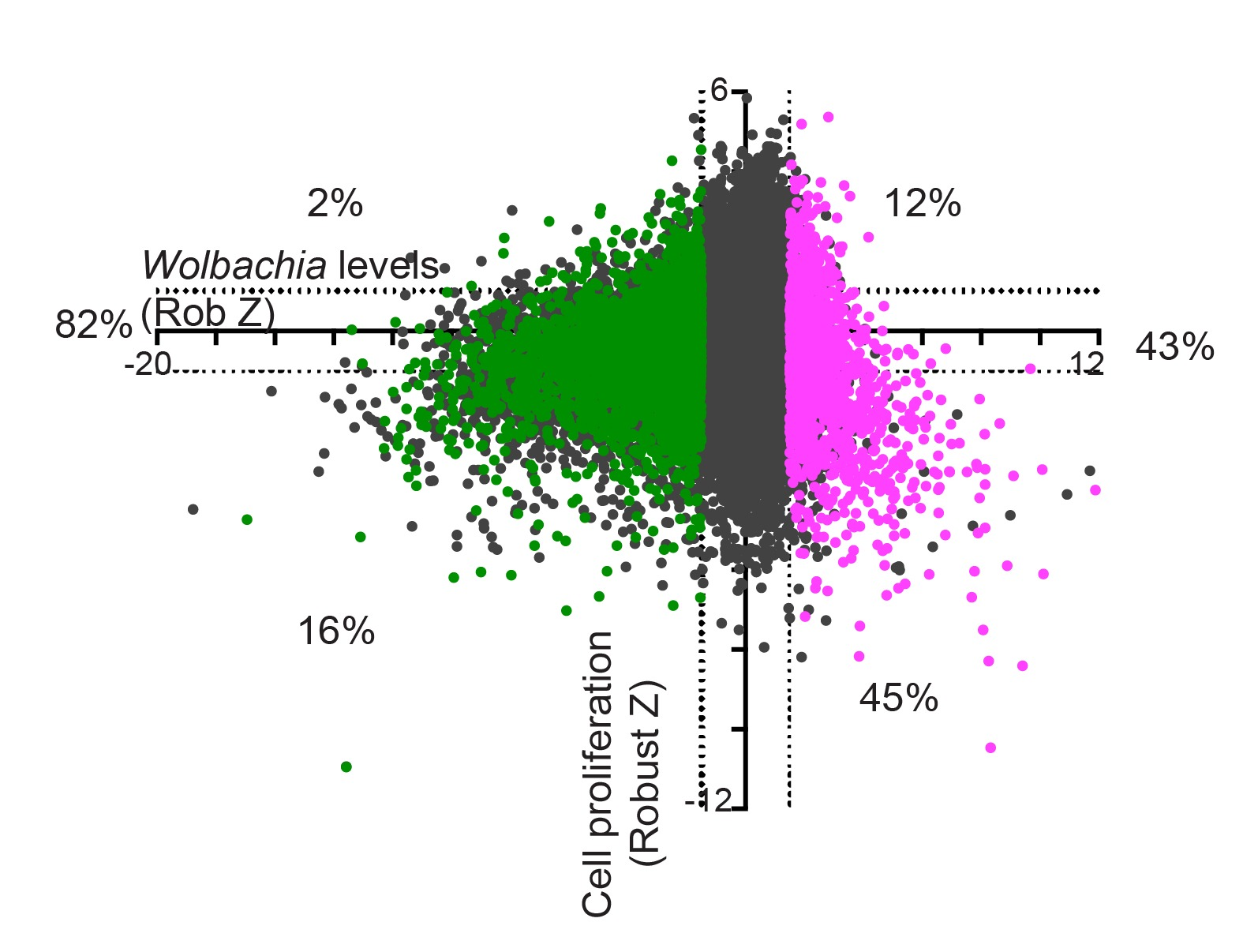

Supplement: S9 Fig — Whole genome comparison of host gene knockdown effect on Wolbachia levels relative to Drosophila cell proliferation. Gene amplicons that significantly decreased Wolbachia levels are represented in green, significant increases in Wolbachia levels are represented in magenta. Each dot represents a single DRSC amplicon in the primary screen, thus every DRSC amplicon is represented at least 3 times as the screen was performed in triplicate. For genes that significantly decreased Wolbachia levels, 2% significantly increased cell proliferation (robZ >1), 82% did not have a significant effect, and 16% significantly decreased cell proliferation (robZ <-1). For genes that significantly increased Wolbachia levels, 12% significantly increased cell proliferation, 43% had no effect, and 45% significantly decreased cell proliferation (robZ<-1). For listing of dsRNA amplicon comparison of changes in Wolbachia levels and cell proliferation see S7 Table). (TIF) [file ppat.1007445.s009.tif]

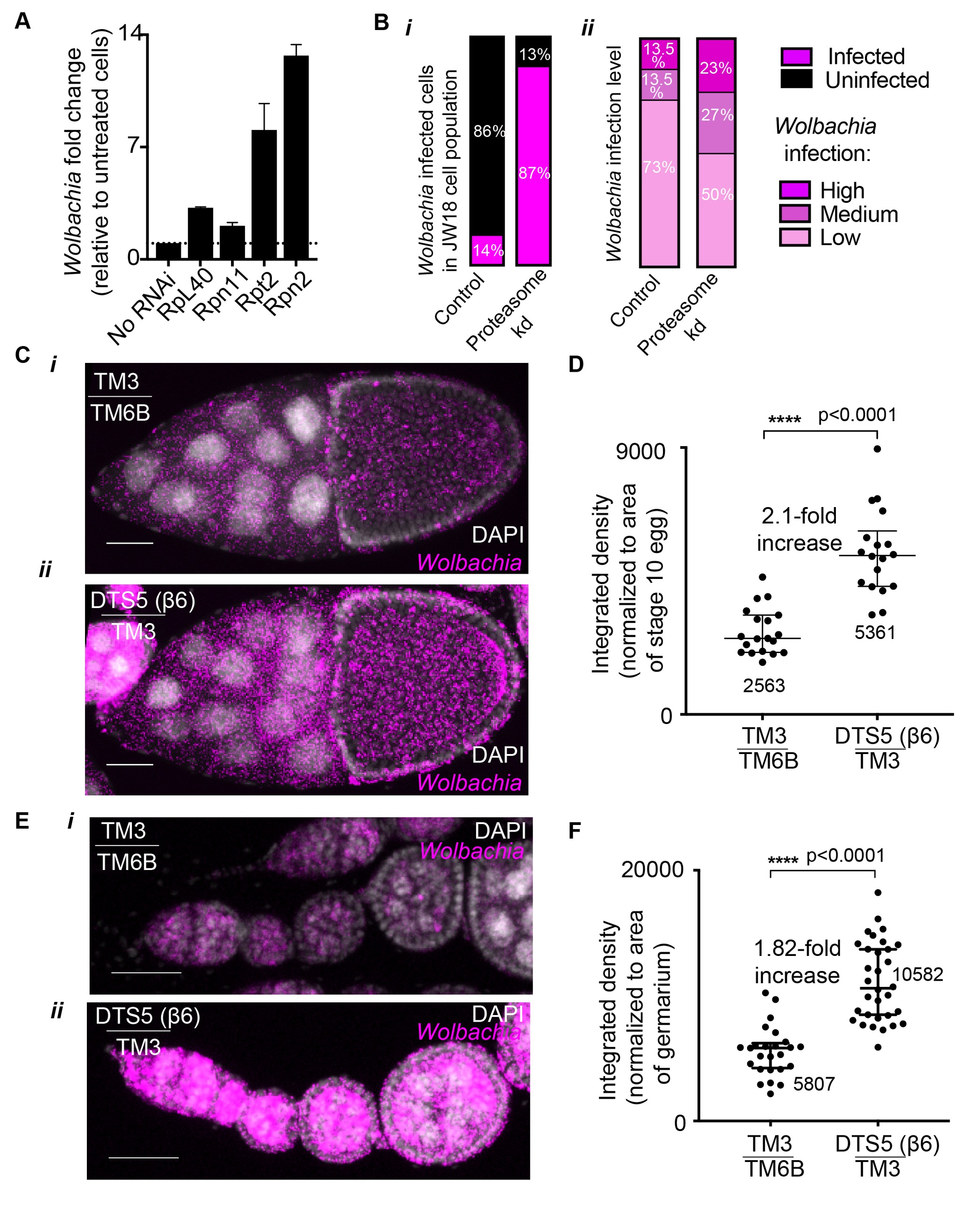

Supplement: S10 Fig — (A) Validation of proteasome network by RNAi in the JW18 cell line. Representative genes were validated using dsRNA amplicons targeting unique regions of each gene. Effects on Wolbachia levels were assessed quantitatively by DNA qPCR measuring the number of Wolbachia genomes using wspB copy number relative to the Drosophila gene RpL11 copy number to represent host cell nuclei. Network validation is relative to untreated JW18 cells and the positive control RpL40 RNAi knockdown is included for reference. (B) Classification of the level of Wolbachia infection within infected cells of the JW18 cell population under control and proteasome (Rpn2) knockdown conditions. (C,E) Wolbachia-infected stage 10 Drosophila egg chambers (C) and germaria (E) of control sibling (TM3/TM6B) and temperature sensitive proteasome mutant sibling (DTS5/TM3) at the restrictive temperature. (D,F) Quantification of integrated density of the Wolbachia FISH probe in stage 10 egg chambers (D) and germaria (F) collected from 15–25 Drosophila ovary pairs for each genotype. Differences between control and mutant siblings are statistically significant (Mann Whitney, p<0.0001). (TIF) [file ppat.1007445.s010.tif]

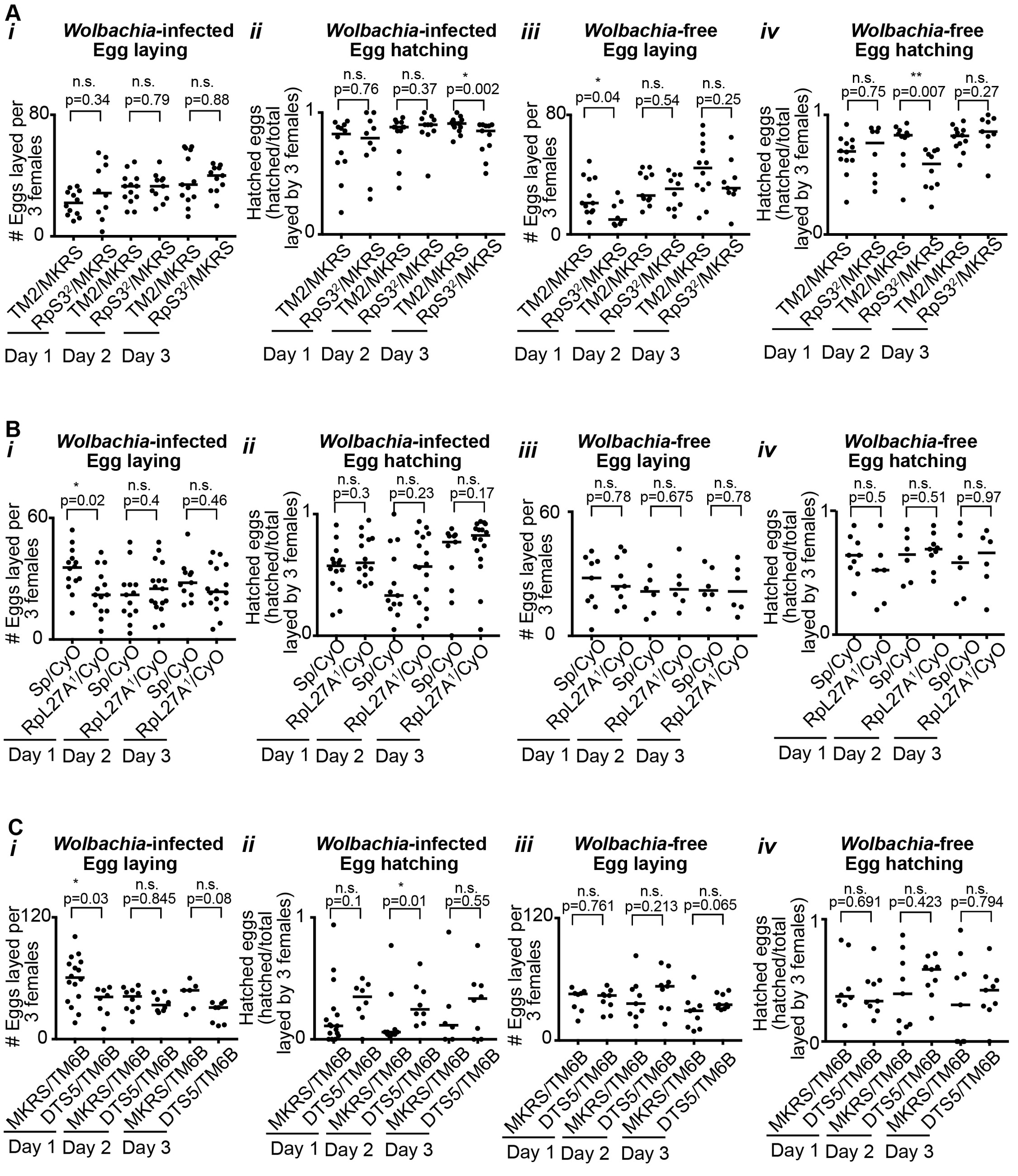

Supplement: S11 Fig — For fecundity testing, we collected the following Wolbachia-infected virgin females crossed to wild-type males: RpS32/MKRS and TM2/MKRS (control sibling) (Ai,ii), RpL27A1/CyO and sp/CyO (control sibling) (Bi,ii), and DTS5/TM6B and MKRS/TM6B (control sibling) (Ci,ii). We also collected the following Wolbachia-free virgin females crossed to wild-type males for fecundity testing: RpS32/MKRS and TM2/MKRS (control sibling) (Aiii,iv), RpL27A1/CyO and sp/CyO (control sibling) (Biii,iv), and DTS5/TM6B and MKRS/TM6B (control sibling) (Ciii,iv). For each genotype between 6–17 ‘3x1’-matings were set up. 24 hours later total eggs layed per 3 females were counted (Ai, iii, Bi, iii, Ci, iii) and scored for hatching (Aii, iv, Bii,iv, Cii, iv). Each ‘3x1’ mating result is represented as a single point of the graphs. See Materials and Methods for details on reciprocal crossing scheme and fecundity testing design. Statistical significance was determined using non-parametric Mann Whitney test. (TIF) [file ppat.1007445.s011.tif]

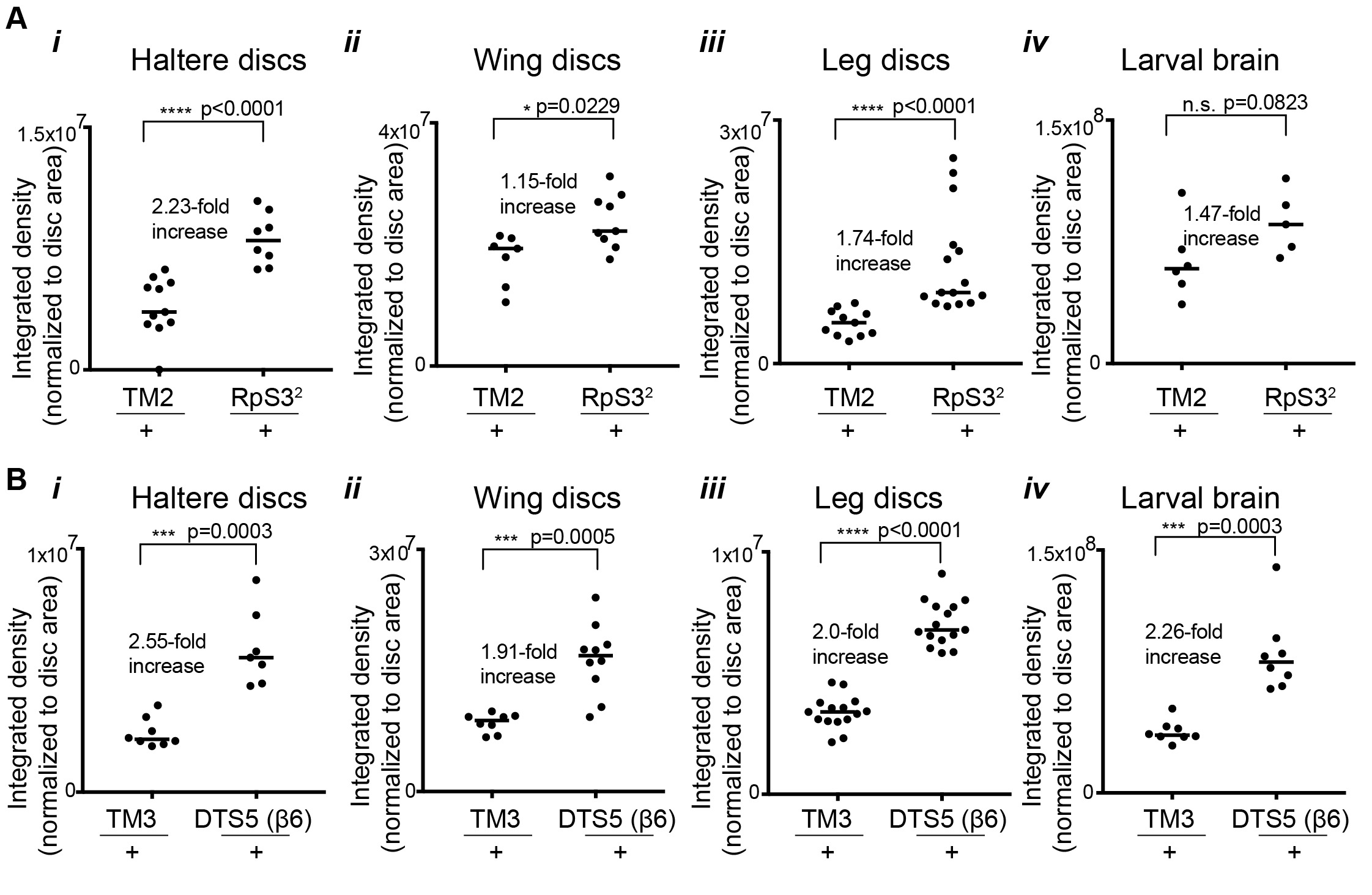

Supplement: S12 Fig — Wolbachia levels in larval imaginal discs of heterozygous ribosomal (A) mutant larvae (RpS32/+) and control larvae (TM2/+) as well as proteasomal (B) mutant larvae (DTS5/+) and control larvae (TM3/+) were assessed by Wolbachia 23s rRNA probe quantification as described for ovarian and testis tissues. Imaginal discs for halteres (i), wings (ii), legs (iii), and the larval brain (iv) were assessed. Individual dots represent individual discs measured. Statistical significance was assessed by non-parametric Mann Whitney test). (TIF) [file ppat.1007445.s012.tif]

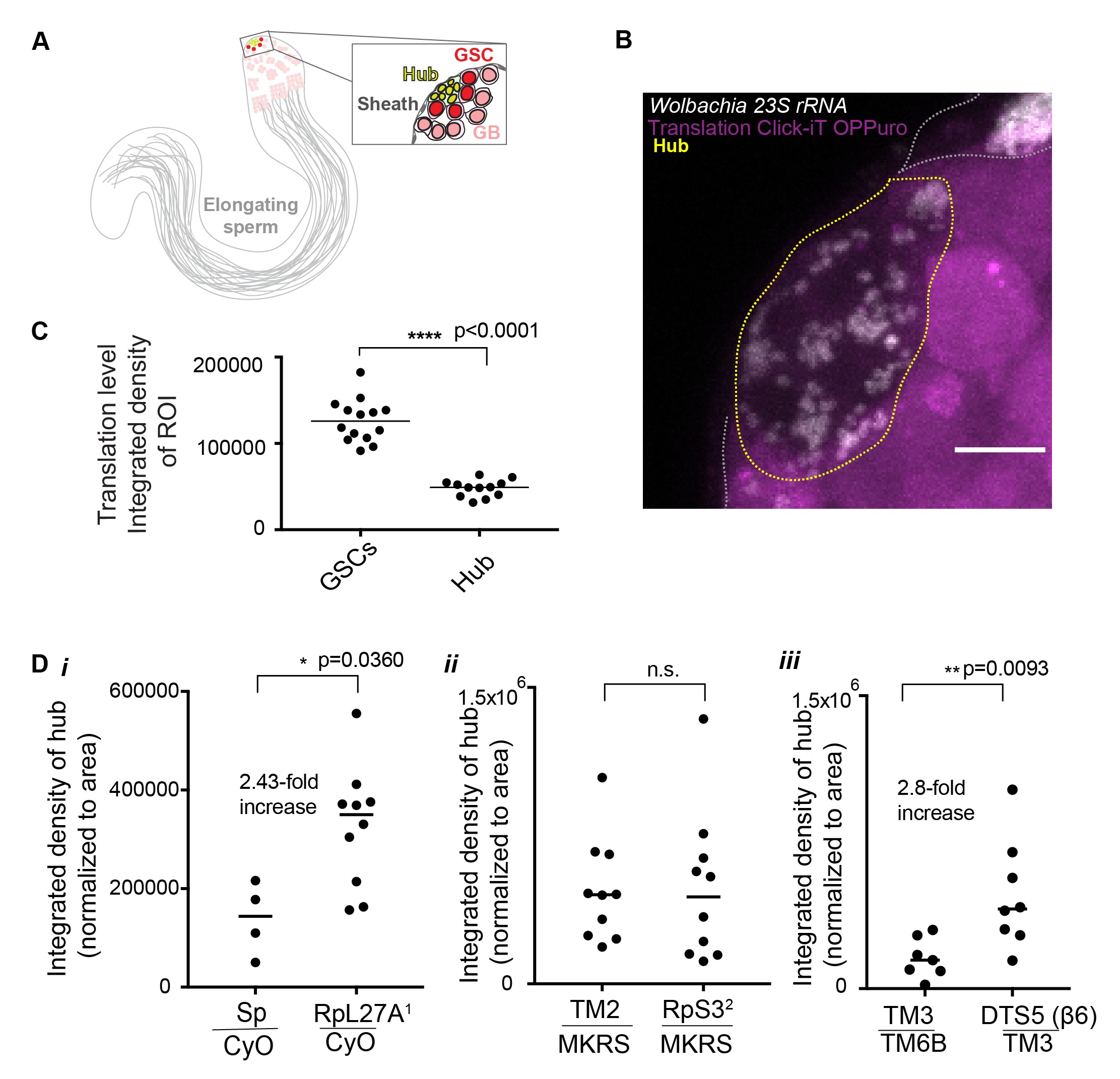

Supplement: S13 Fig — (A) The Drosophila testis illustration highlighting the stem cell niche (hub) at the tip of the testes surrounded by and directly contacting the germline stem cells (GSCs) (dark red). GSCs divide perpendicular to the hub to maintain one GSC that touches the hub and one daughter cell (light red) that matures into a developing sperm. The testis is surrounded by sheath cells (grey). (B) Projected Z-stack of a Wolbachia-infected Drosophila testis hub (yellow outline). Testes were treated with Click-iT OPPuro to fluorescently label newly synthesized proteins (purple) and Wolbachia are labeled by FISH (white). Wolbachia mainly occupy the hub niche and sheath cells (grey outline) which have low levels of translation as measured by Click-iT OPPuro assay (purple). Scale bar represents 5μm. (C) Quantification of HPG OPPuro assay fluorescent signal for protein synthesis measured in ImageJ as integrated density in the hub compared to surrounding GSCs. Lines represent median. (D) Quantification of Wolbachia levels in testis hubs of ribosomal mutants (RpL27A1/CyO (i), RpS32/MKRS (ii)) compared to sibling controls (Sp/CyO (i) TM2/MKRS (ii) respectively) and proteasomal mutant (iii) (DTS mutant compared to sibling control) by integrated density measurement of 23s rRNA Wolbachia FISH probe using Fiji software to outline and measure within the testis hub. Each dot represents an individual testis. Lines represent median. Statistical significance was measured using non-parametric Mann Whitney test. (TIF) [file ppat.1007445.s013.tif]

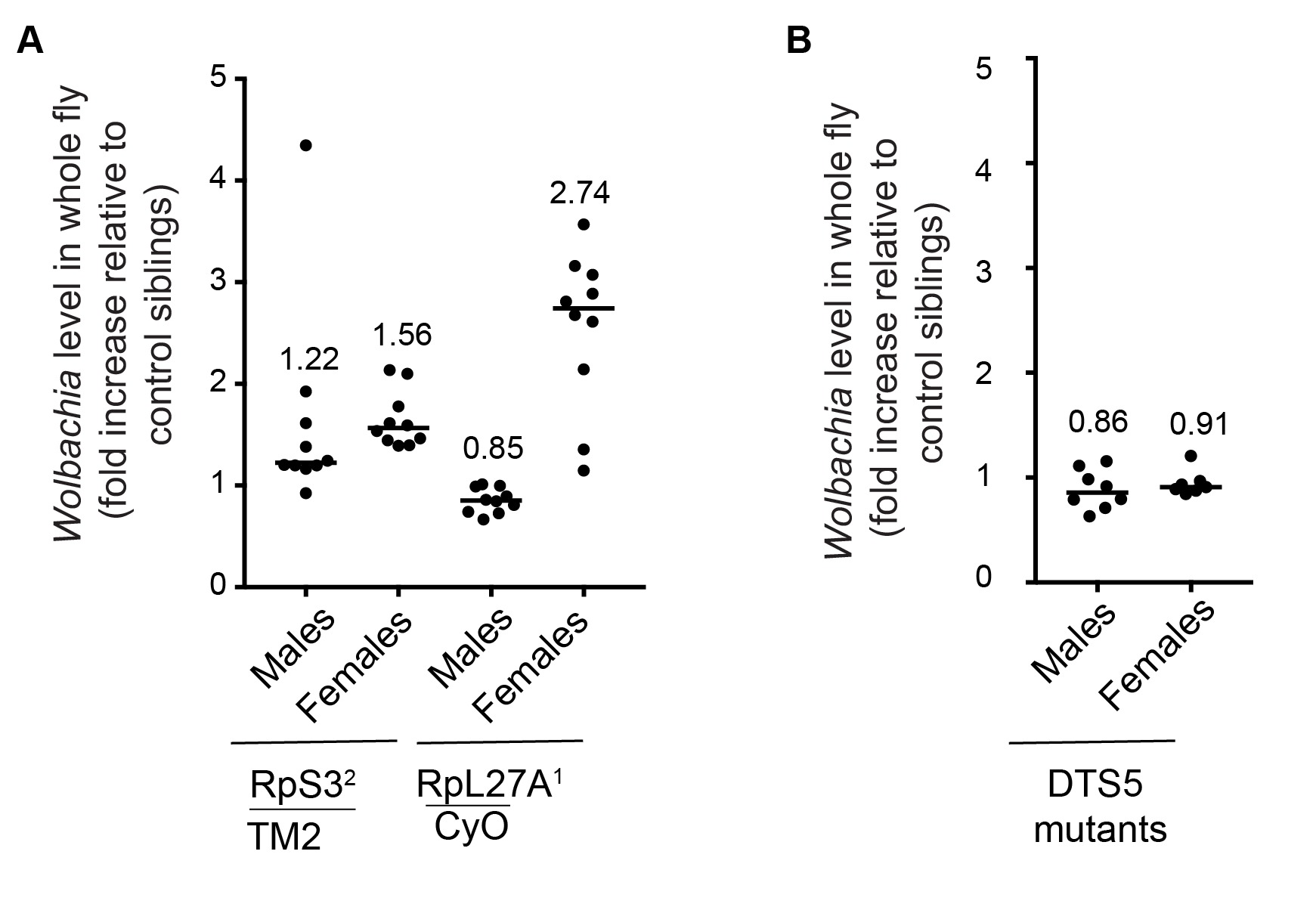

Supplement: S14 Fig — Wolbachia levels in whole flies were assessed by DNA qPCR analysis for ribosomal (A) and proteasomal (B) mutants relative to control siblings. Male and female individual whole flies were processed for gDNA extraction and subjected to DNA qPCR. Each dot represents an individual fly. Lines represent median of each genotype. The effect on Wolbachia level is represented as a fold-increase relative to control siblings. (TIF) [file ppat.1007445.s014.tif]

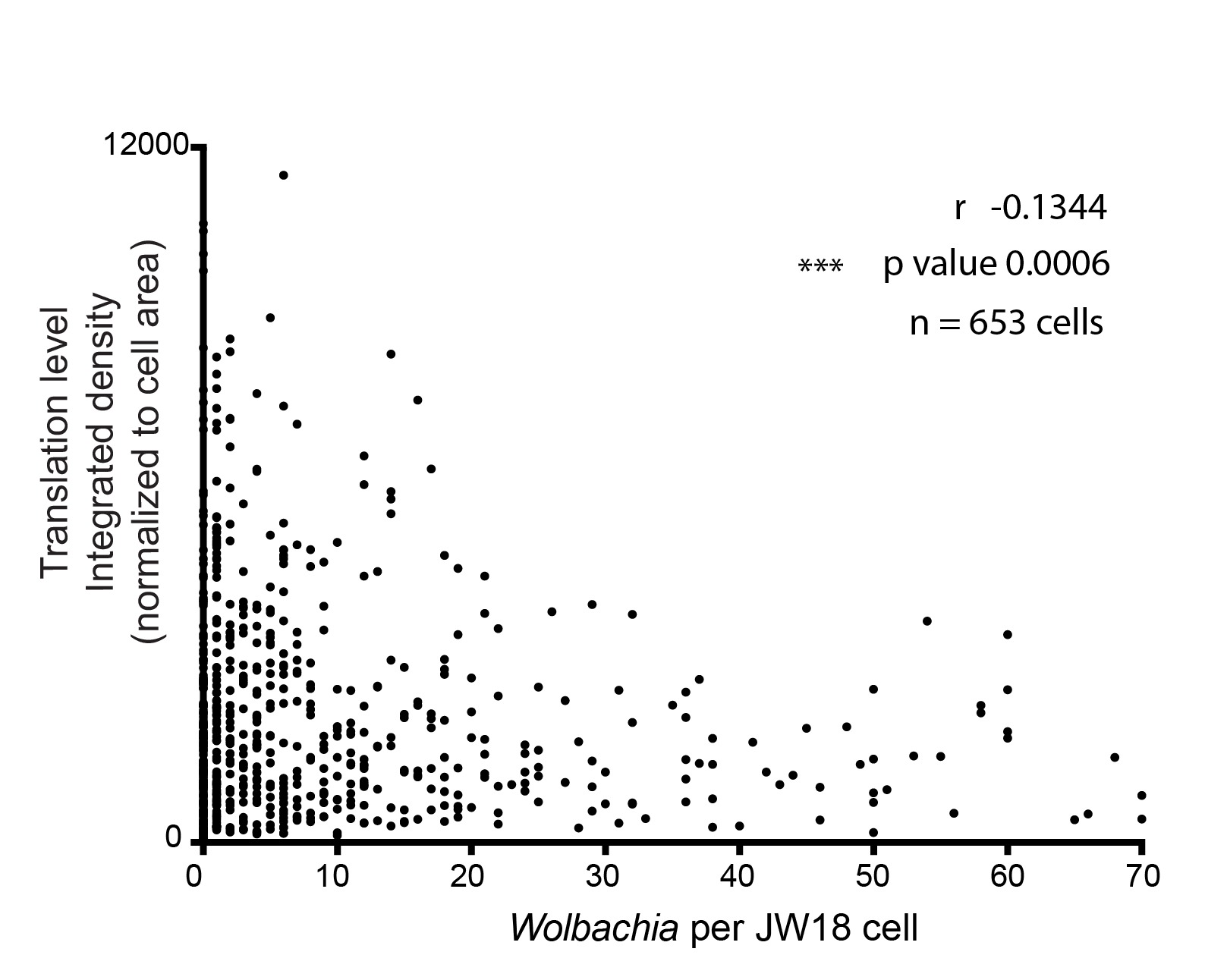

Supplement: S15 Fig — Translation level was measured in 653 individual cells of a JW18 population in the same manner as Fig 7C and 7D. Translation level in host cells is negatively correlated to Wolbachia level within individual cells (r = -0.1344, p = 0.0006, Pearson’s correlation). (TIF) [file ppat.1007445.s015.tif]

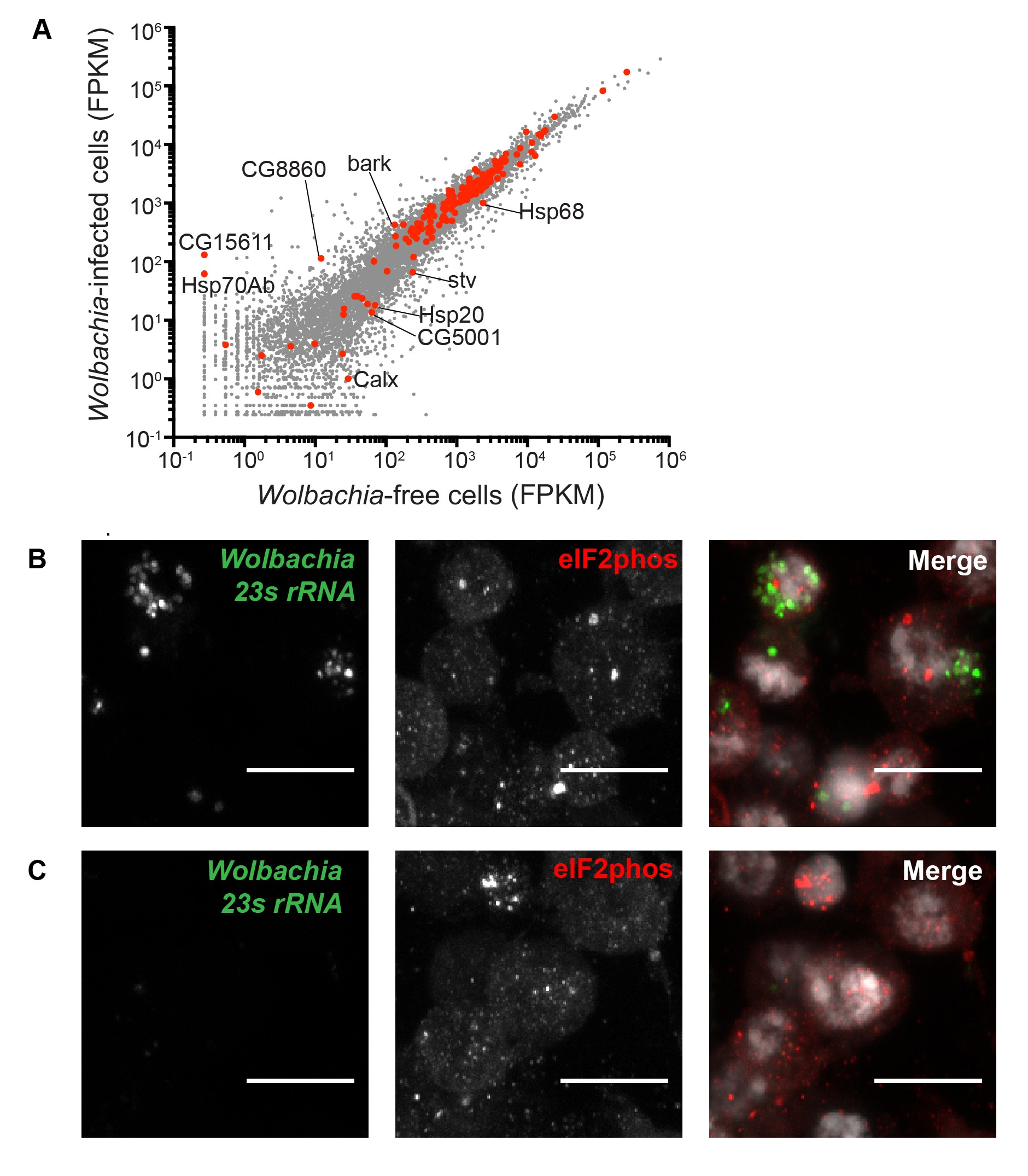

Supplement: S16 Fig — (A) RNAseq analysis on Wolbachia-infected JW18 cells compared to JW18DOX cells highlighting stress related genes’ expression in response to Wolbachia infection in Wolbachia-infected cells. (B, C) JW18 cells do not show altered eIF2 phosphorylation antibody staining compared to Wolbachia-free cells. (TIF) [file ppat.1007445.s016.tif]
